# Linking Perceived Hospital-Generated Content to Psychological Well-Being Through Integrated Cognitive and Emotional Resilience Mechanisms Among Emerging Adults

**DOI:** 10.3390/ijerph23060718

**Published:** 2026-05-28

**Authors:** Audrey Hamdoyo, Ferdi Antonio, Janeline Rivana Sefty Tengor

**Affiliations:** Department of Hospital Administration, Pelita Harapan University, Jakarta 12930, Indonesia; hamdoyoaudrey@gmail.com (A.H.); janeline.tengor@gmail.com (J.R.S.T.)

**Keywords:** cyberchondria, neuroticism, digital health literacy, hospital-generated content, psychological well-being, clinical psychology, young adults, emerging adults

## Abstract

**Highlights:**

**Public health relevance—How does this work relate to a public health issue?**

- The digital information ecosystem has emerged as a critical social determinant of mental health for Generation Z as a vulnerable population.
- Rising rates of digitally mediated health anxiety and cyberchondria among adolescents and young adults present an urgent global public health challenge.

**Public health significance—Why is this work of significance to public health?**

- The findings suggest that hospital-generated digital content has the potential to serve as a valuable resource for community-level mental health promotion.
- It outlines a conceptual framework indicating that scientifically reliable and humanizing digital messaging supports adaptive coping mechanisms.

**Public health implications—What are the key implications or messages for practitioners, policy makers and/or researchers in public health?**

- Healthcare providers, such as private and public hospitals, are encouraged to employ mental health professionals to clinically screen public-facing content and ensure it promotes autonomous health management.
- Health policymakers and government agencies are encouraged to co-author digital health policy guidelines that facilitate ‘digital environmental engineering,’ transforming institutional content into a tool for mental health advocacy.

**Abstract:**

Background: Hospital-generated digital health content (HGC) represents an emerging psychosocial influence on young adults’ internalizing trajectories. This study examined how perceived HGC variables may associate with psychological well-being (PWB) through cognitive-behavioral and affective mediators, with neuroticism (NEU) moderating these associations. Methods: In a cross-sectional online survey, 248 Indonesian respondents (born 1997–2012) who regularly consumed HGC completed validated and adapted instruments. The data were analyzed using PLS-SEM. Results: Perceptions of HGC function as key social-cognitive correlates of PWB in emerging adults. Perceived scientific reliability (PSR) and peace of mind (PEM) are positively associated with digital health literacy (DHL), cyberchondria resilience (CYR), and emotional regulation strategies (ERS). Conversely, humanizing value (HMV) is linked with a potential “comfort-without-competence” trajectory. The CYR construct emerged as the primary mediating mechanism for PWB, whereas NEU was found to weaken the positive association between DHL and PWB. Conclusion: These results suggest that HGC is associated with enhanced PWB by facilitating information competence, reducing compulsive health-seeking, and supporting emotional regulation, though emotionally warm formats may be linked with lower literacy development. The personality trait NEU selectively moderates cognitive pathways while leaving affective and behavioral pathways intact, suggesting a need for risk-stratified approaches. By providing clinically screened content that integrates cognitive-behavioral scaffolding, healthcare institutions can support early psychological prevention and self-regulation.

## 1. Introduction

### 1.1. Background

By January 2025, Generation Z (born 1997–2012) comprised 22.9% of the global population [1] and represents a digitally native cohort that routinely accesses health information via mobile devices and social media. This immediateness, intensified during the COVID-19 pandemic, enables rapid exposure to medical concepts that medical professionals previously mediated [2]. This accelerated circulation of health knowledge advances informed self-care while simultaneously heightening exposure toward fragmented or alarming material that promotes reassurance-seeking and health anxiety among digitally active youth [3,4]. When this behavior becomes compulsive, it manifests as cyberchondria, defined as a multidimensional pattern characterized by excessive and repetitive online searches for health-related information that ultimately escalates rather than alleviates health anxiety [5].

According to the World Health Organization (WHO), emotional disorders are common among late adolescents, as evidenced by global prevalence rates of 5.3% for anxiety and 3.4% for depression within the 15–19 age group [6]. A systematic analysis of the Global Burden of Diseases Study (2021) also reports a mental disorder rate of roughly 23.1 per 1000 for the 20 to 24 age group [7]. This reality is reflected in Indonesian national statistics, as the prevalence of mental health conditions affects an estimate of 6,1% of Indonesians over the age of 15 [8]. To address the widening mental health gap in Indonesia, recent local studies increasingly advocate for community-based promotion and preventive interventions to support psychological well-being [9,10].

Evidence suggests that Generation Z faces heightened vulnerability to anxiety symptoms, potentially linked to pervasive digital access and lifelong immersion in online environments [11]. A large-scale Chinese study conducted during the COVID-19 outbreak revealed that intensive health information searching was significantly correlated with generalized anxiety and depressive symptoms, particularly among youth populations [12]. Consistent with these findings, a systematic review from 2023 reported that excessive screen time is positively associated with impaired adolescent well-being, with anxiety-related outcomes prominently represented [13]. These dynamics constitute challenges to the objectives of mental health promotion and prevention frameworks, which seek to address psychosocial risk factors through population-level strategies [14]. Through amplifying distress and sustaining maladaptive information-seeking cycles, digital health environments complicate population-level efforts to reduce morbidity and underscore the need for evidence-based, harm-minimizing communication strategies that support resilience and adaptive coping.

In response to changing media consumption patterns among youth, it is essential for healthcare stakeholders to invest in community-level synergy and preventive measures. One of the primary stakeholders within the national healthcare domain includes hospitals. Nowadays, hospitals have increasingly adopted digital communication strategies to advocate for health [15]. However, emerging research suggests that hospital-generated content (HGC) is susceptible to erosion of its educational value within entertainment-driven social media environments [16,17]. This erosion is particularly alarming because hospitals, as extensions of clinical systems, possess a greater inherent potential to deliver trustworthy health information compared to independent creators operating outside formal healthcare systems [18,19]. The predominance of short-form video formats encourages affective engagement yet constrains higher-order cognitive processing necessary for reflective learning [20,21].

Emerging adulthood, defined as ages 18–29 [22], represents a critical period characterized by a neurological imbalance that leaves individuals more susceptible to environmental stressors as their capacity for reasoned problem-solving continues to develop alongside impulsive centers [23]. Hospital-generated content should therefore provide clinical scaffolding by prioritizing empathetic engagement and psychosocial safety over generic prescriptive guidelines. By functioning as a credible and nuanced resource, such content supports the emerging adults’ transition toward independent and sophisticated health information processing. Accordingly, the central research question is: To what extent does HGC consumption enhance psychological well-being (PWB) through psychological mediators such as digital health literacy, cyberchondria resilience, and emotion regulation strategies, and does neuroticism moderate these relationships? To address these questions, a deductive, cross-sectional survey was conducted among Generation Z using a conceptual model grounded in current literature. By integrating these variables, the study provides an original perspective that positions HGC as an emerging mental health determinant, specifically examining the protective role of cyberchondria resilience within cognitive-affective processing and the moderating influence of neuroticism in Generation Z.

### 1.2. Present Studies and Hypotheses Development

Current studies utilize the theory of communicative action [24,25] to evaluate the validity of institutional messaging and social cognitive theory [26] to examine the development of self-regulatory agency. These perspectives converge in Diener’s model of well-being [27,28], which frames psychological health as a functional balance between cognitive life satisfaction and positive affect. Recent findings highlighted that online health information use (including hospital-generated content; HGC) and mental health outcomes are linked through cognitive and emotional mechanisms [2,29,30,31]. A key cognitive factor in this pathway is digital health literacy (DHL), defined as the capacity to locate, evaluate, and meaningfully apply health information [31]. The way individuals regulate their emotional responses to any form of health information, termed as emotion regulation strategies (ERS), also appears to be essential in determining whether such exposure supports mental health outcomes or exacerbates distress [32,33].

Research on DHL and seeking health information online supports digital empowerment among young adults [31,34]; however, this same behavior may become a liability when excessive searching transforms into cyberchondria [5,35] unless resilience (CYR) buffers the pathway. The existing literature also links cyberchondria to emotion-regulation difficulties, and the ability to manage reassurance-seeking appears to be a key factor in maintaining behavioral balance [36,37,38], yet the CYR construct remains underexplored. Together, these constructs capture complementary cognitive and affective pathways linking HGC consumption to mental health outcomes.

Perceived scientific reliability (PSR) is defined as a cognitively grounded dimension of credibility concerned with truthfulness and transparency [39]. Several studies show that perceived trust [29,40,41], credibility [40], altruistic motivation [41], and information quality [29] influence how users respond to online health content. Recent empirical evidence also shows that higher trust and credibility in health information sources are associated with higher DHL [42]. A previous review also concluded that authority and content credibility are key determinants of trust in online health information [40], suggesting that stronger credibility judgments can improve users’ ability to differentiate reliable information and help reduce maladaptive reactions. Additionally, distrust in health information has also been found to correlate with higher health anxieties, exhibiting characteristics similar to cyberchondria, as well as negative emotional reactions [43]. Nevertheless, the direct evidence connecting credibility to improved CYR and ERS remains sparse and highly context-sensitive across different digital platforms and user characteristics [44]. Accordingly, higher PSR is hypothesized to positively associate with DHL, CYR, and ERS (H1–H3).

Humanizing value (HMV) captures how HGC evokes personal, empathetic, and relational responses across sensory, emotional, cognitive, and behavioral domains rather than appearing purely institutional [45]. Such empathetic and patient-centered messaging is known to deepen trust and engagement, fostering attentive interaction with information, which may promote DHL [46]. At the same time, empathetic cues may buffer emotional distress and reduce the uncertainty that drives compulsive reassurance-seeking behaviors; consistent evidence shows that empathy-based interactions lessen anxiety and strengthen coping resources [47], denoting a protective role that strengthens resilience against cyberchondria. Moreover, humanizing elements also support self-reflection and meaning-making, mechanisms shown in narrative medicine to enhance emotional regulation [48]. On this basis, HMV is projected to positively associate with DHL, CYR, and ERS (H4–H6).

Narrative transportation (NRT) describes the degree to which a person becomes mentally and emotionally immersed [49]. In the context of health communication, narrative frameworks elevate engagement and motivation [48,50], indirectly improving DHL by prolonging attention and sustaining suitable conditions for information processing. Empirical research demonstrates that narrative engagement cultivates social-cognitive skills like theory of mind and empathy [51], which support emotion regulation and enhance resilience against anxiety from vague health information [52]. Evidence from narrative exposure therapy shows that structured narrative processing can regulate emotions and alleviate distress, suggesting that well-designed health narratives may similarly reinforce resilience against cyberchondria [53]. Therefore, NRT is expected to positively associate with DHL, CYR, and ERS (H7–H9).

Peace of mind (PEM) is conceptualized as the psychological comfort and assurance individuals experience when they perceive HGC as reliable, user-friendly, and committed to their sustained welfare [54,55]. Although explicit empirical evidence linking peace of mind to subsequent gains in DHL remains limited, related research on emotional safety, trust, and learning suggests that affective calm may enable deeper cognitive engagement with health information [56]. Peace of mind (PEM) is therefore posited as a foundational enabling condition that facilitates DHL, CYR, and ERS (H10–H12) by reducing defensive processing and cognitive overload.

Following the aforementioned evidence and contextual background, the hypotheses of this study are:

**H1.**  *Perceived scientific reliability of hospital-generated health content positively associates with digital health literacy.*

**H2.**  *Perceived scientific reliability of hospital-generated health content positively associates with emotional regulation strategies.*

**H3.**  *Perceived scientific reliability of hospital-generated health content positively associates with cyberchondria resilience.*

**H4.**  *Humanizing value of hospital-generated health content positively associates with digital health literacy.*

**H5.**  *Humanizing value of hospital-generated health content positively associates with cyberchondria resilience.*

**H6.**  *Humanizing value of hospital-generated health content positively associates with emotional regulation strategies.*

**H7.**  *Narrative transportation derived from hospital-generated health content positively associates with digital health literacy.*

**H8.**  *Narrative transportation derived from hospital-generated health content positively associates with cyberchondria resilience.*

**H9.**  *Narrative transportation derived from hospital-generated health content positively associates with emotional regulation strategies.*

**H10.**  *Peace of mind elicited by hospital-generated health content positively associates with digital health literacy.*

**H11.**  *Peace of mind elicited by hospital-generated health content positively associates with cyberchondria resilience.*

**H12.**  *Peace of mind elicited by hospital-generated health content positively associates with emotional regulation strategies.*

As HGC seeks to inform and reassure, evaluating its comprehensive relationship with well-being clarifies whether such content alleviates or exacerbates anxiety and stress in young users. Population-level mental health approaches focus on prevention, early intervention, and adaptive functioning across groups [57], highlighting the need for universal measures of mental health outcomes. Psychological well-being (PWB) fulfills this role because it represents emotional balance, positive affect, and healthy self-evaluations [27,28,58], making it applicable across cultures and developmental stages [59]. Empirical evidence showing that Generation Z is particularly vulnerable to mental health strains associated with intensive digital media use [60] reinforces the relevance of measuring psychological well-being (PWB) in this cohort. Positioning PWB as the primary outcome enables this study to assess mental health as positive functioning rather than merely the absence of pathology, aligning with public mental health objectives [61].

As previously illustrated, higher levels of DHL are expected to enhance PWB outcomes, as found by multiple studies on young adults [31,62] (H13). Cyberchondria resilience (CYR) is likewise theorized to help improve PWB (H14) by limiting anxiety amplification and maladaptive reassurance-seeking behaviors [63]. Effective ERS are similarly hypothesized to support positive affect and reduce distress subsequent to health information exposure (**H15**) through multiple established mechanisms [32,33].

**H13.**  *Digital health literacy positively associates with psychological well-being.*

**H14.**  *Cyberchondria resilience positively associates with psychological well-being.*

**H15.**  *Emotional regulation strategies positively associate with psychological well-being.*

Finally, personality traits are included as a moderating factor because they emerge from multiple genetic and environmental determinants that often lie outside the individual’s immediate control [64]. Evidence shows that individuals with high neuroticism (NEU) display amplified stress reactivity, diminished emotion regulation, and threat-biased attention, thereby weakening the downstream effect of psychological resources on well-being [65]. Neuroticism (NEU) is a well-established predictor of broad psychological vulnerability and common mental disorders [66,67,68]. Behavioral studies have shown that individuals high in NEU tend to report greater emotional reactivity and problematic internet behaviors, including patterns of excessive health-searching and cyberchondria [69,70]. Evidence from twin studies shows that NEU exhibits higher heritability compared to other personality traits [71], which helps explain its stability and importance as a moderator. Clinically, these moderation dynamics mirror vulnerability models where NEU heightens threat perception, negative affect, and coping deficits [72]. However, its specific role within digital health information environments remains underexplored. Rather than exerting uniform effects across pathways, NEU is theorized to weaken the relationships between CYR and ERS with PWB (H16–H18).

**H16.**  *Neuroticism moderates the relationship between digital health literacy and psychological well-being, s* *uch that this relationship weakens at higher levels of neuroticism.*

**H17.**  *Neuroticism moderates the relationship between cyberchondria resilience and psychological well-being, s* *uch that this relationship weakens at higher levels of neuroticism.*

**H18.**  *Neuroticism moderates the relationship between emotional regulation strategies and psychological well-being, s* *uch that this relationship weakens at higher levels of neuroticism.*

## 2. Materials and Methods

### 2.1. Study Design and Sampling Technique

This cross-sectional study used an online quantitative survey. Participants were selected through purposive sampling using the following inclusion criteria: birth year between 1997 and 2012, and active consumption of hospital-generated health content. Exclusion criteria comprised any history of clinician-diagnosed psychotic disorder and/or any past or current self-reported psychotic symptoms. The recruitment process yielded a total of 248 responses that met the eligibility criteria, comfortably exceeding the minimum sample size. The sample calculation was performed in G*Power^®^ 3.1 using a linear multiple regression model (R^2^ deviation from zero) with eight predictors, α = 0.05, power = 0.90, and an assumed medium effect size (f^2^ = 0.15), which indicated a minimum required N of 136. This method was selected because it estimates the sample needed to detect whether multiple predictors jointly account for a non-zero proportion of outcome variance [73], aligning with the study’s multivariate objectives.

### 2.2. Conceptual Framework

The theoretical foundation for modern youth transitions began with Erik Erikson’s introduction of the psychosocial moratorium as a period of prolonged adolescence dedicated to identity exploration [74,75]. This framework was subsequently advanced by Jeffrey Arnett, who established emerging adulthood as a unique developmental phase for individuals in their late teens and twenties [22]. During this stage, individuals remain highly sensitive to environmental stimuli as they navigate the transition from parental co-regulation toward autonomous self-regulation [76]. Consistent with these developmental needs, supportive social interactions function as essential building blocks while negative experiences elevate the risk of psychological distress [77]. Consequently, this study conceptualizes health information as a modern social stimulus that influences the well-being of emerging adults through both its informational quality and its interpersonal tone.

The architecture of health messaging is multidimensional, offering a broad matrix of content variables, especially within the specific context of HGC. Drawing on Habermas’ theory of communicative action [39], this study organizes clinically relevant features into four functional dimensions: truth, sincerity, appropriateness, and understandability. Translating these into the context of HGC, truth is operationalized as perceived scientific reliability (PSR), sincerity as humanizing value (HMV), appropriateness as peace of mind (PEM), and understandability as narrative transportation (NRT). These four antecedent variables form the foundation of the proposed model, functioning as content characteristics that activate the psychological mechanisms examined in this research.

The theoretical framework follows the principles of social cognitive theory, which explains behavior as a product of interactions between environmental influences, internal psychological processes, and individual traits [78]. Within this context, HGC functions as the external stimulus that provides informational and emotional cues, while DHL, CYR, and ERS represent the internal cognitive and emotional mechanisms through which individuals interpret and manage health information. Although cognitive reappraisal [79] and expressive suppression [80,81] are typically treated as distinct strategies [79,82], this study operationalizes them as a unified construct representing overall regulatory effort. In the context of consuming alarming digital health information, individuals often mobilize multiple coping mechanisms simultaneously to manage acute anxiety. By treating these two strategies as a singular factor of regulatory mobilization, this study focuses on the sheer presence of emotional management efforts rather than comparing their independent mechanisms. Neuroticism (NEU) is introduced as the personal factor that moderates these relationships by shaping stress sensitivity, emotional reactivity, and interpretive tendencies in digital settings.

Adopting psychological well-being as the primary clinical outcome is grounded in evidence that it serves as a foundational marker of mental health and a vital protective factor against the development of long-term psychopathology [83]. Among the various conceptualizations of well-being, Diener’s subjective well-being framework is the most directly applicable because it emphasizes emotional balance and satisfaction with life [27]. Das et al. expanded this perspective by demonstrating that subjective well-being is shaped by factors that influence behaviors, cognitive evaluations, personal goals, and emotional functioning [84]. In accordance with developmental theories of transition, the model illustrates how institutional digital platforms contribute to the development of behavioral tendencies of emerging adults. In this context, the mediators function as internal regulatory processes that determine how young users evaluate health threats, while the outcome of psychological well-being serves as a measure of adaptive emotional functioning in a digital society.

**Figure 1:**
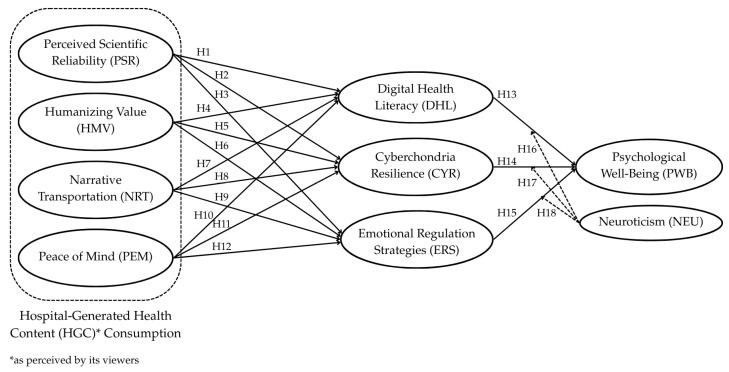
Research framework.

Drawing deductively from foundational theories and prior empirical studies, this study proposes three mediating psychological processes (DHL, CYR, and ERS) and one moderating variable (NEU) to explain how HGC may shape PWB, as shown in Figure 1.

Taken together, this framework integrates communicative action theory [39], social cognitive theory [78], and subjective well-being theory [27,84] to evaluate how institutional digital ecosystems influence population-level mental health. By mapping these multivariate pathways, the study advances prior research by shifting the focus from isolated variables toward a comprehensive understanding of how digital environments function as determinants of psychological outcomes.

### 2.3. Definition of Variables

To ensure uniformity in conceptual understanding between the theory and the measurement instruments, Table 1 presents the operational definitions used to test the hypotheses.

### 2.4. Construct Variables

Measurement items were adapted from established psychometric instrument references through a multi-stage validation process. A sworn translator performed the initial Indonesian translation, while a second professional conducted a back-translation to maintain conceptual equivalence. The instrument then underwent a cognitive review with five members of the target demographic to ensure item clarity and cultural relevance. Content and face validity were subsequently confirmed by an expert panel of three clinicians and researchers. To reduce the risk of common method bias (CMB) and socially desirable responding inherent in self-reported, cross-sectional survey designs, this study implemented multiple procedural remedies in line with the methodological recommendations of Podsakoff et al. (2003) [88]. This survey was administered anonymously through a computer-based platform, with confidentiality explicitly emphasized, as part of a deliberate strategy to reduce social desirability bias. Participants were informed that the questionnaire contained no right or wrong answers and were encouraged to provide honest responses. Together, these integrated measures strengthen construct validity by ensuring that the observed associations reflect genuine psychological relationships rather than measurement artifacts [88].

### 2.5. Data Collection and Screening Procedure

**Figure 2:**
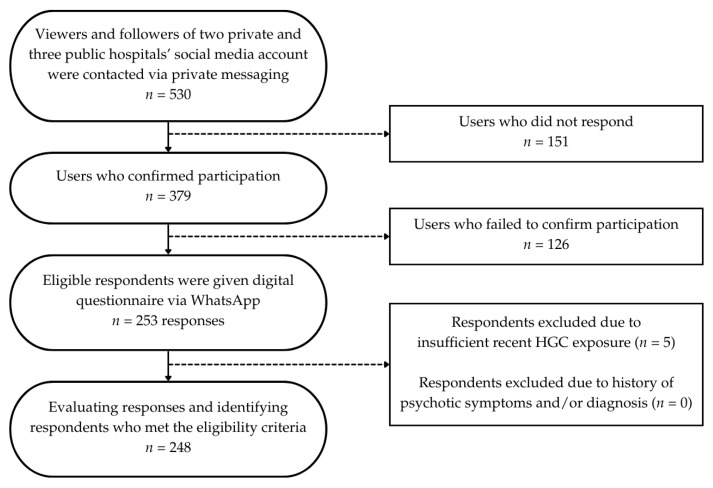
Participant selection flow.

The accessible population was extracted from viewers and followers of social media accounts of two private hospitals and three government hospitals in December 2025 (Figure 2). Candidates were identified by observing public follower lists and interactions on the hospitals’ open social media profiles. Direct institutional clearance from the hospitals was not sought because the recruitment relied entirely on public digital spaces and did not involve proprietary internal records. Each identified user was contacted via private messaging to confirm participation. During this initial contact, the researchers explicitly disclosed independent status and clarified that this study is not affiliated with or endorsed by the selected hospitals. To strictly protect user privacy, the initial message contained only a brief study description and a secure survey link. The questionnaire was then distributed through WhatsApp only to users who provided explicit digital informed consent. To ensure privacy, all messaging histories were deleted immediately after data collection, and no contact identifiers were retained in the final dataset. The resulting sample specifically represents a sub-population of digitally active young adults who are already engaged with health communications online rather than the broader Generation Z demographic.

Respondents then completed a structured screening module documenting their sociodemographic characteristics as well as relevant behavioral and psychosocial factors before proceeding to the questionnaire items. Additionally, two semantic-differential screening items were included to verify that respondents exhibited measurable neuroticism tendencies. Respondents rated themselves on the five-point bipolar adjective scales: “calm and emotionally stable” versus “easily worried and tense” and “rarely a worrywart” versus “easily overly worried.” To ensure the sample reflected the target population, active consumption was operationalized as self-reported engagement with hospital-generated content at least once in the past month. A total of five respondents were removed from the study after screening due to the lack of recent HGC consumption. No respondents disclosed current and/or history of psychotic symptoms. Individuals with non-psychotic clinical diagnoses were retained to adequately assess varying degrees of neuroticism, recognizing that excluding all psychiatric conditions would disproportionately remove young adults at the highest end of the neuroticism scale. This resulted in 248 eligible respondents included in the data analysis (151 female, 85 male, and 12 respondents who preferred to not answer).

### 2.6. Data Analysis

The data results were initially tabulated in Microsoft Excel 2019, then analyzed for both the measurement model analysis and the structural model analysis with SmartPLS4 4.1.1.6 for Windows. PLS-SEM is the preferred method of analysis due to the explanatory predictive orientation of this study, as well as its versatility in handling smaller sample sizes and complex model structures [89]. Using SEM path analysis, the mediating and moderating roles of hospital-generated digital health content, digital health literacy, cyberchondria resilience, and emotional regulation strategies, as well as neuroticism on psychological well-being, were explained. Standardized regression coefficients (β) were used to detect the quantifiers’ direct and indirect effects.

### 2.7. Ethical Consideration

This study protocol has been reviewed and approved by the Institutional Ethics Committee of Universitas Pelita Harapan, as documented in the ethical clearance letter (No. 058/MARS/EC/XII/2025) confirming compliance with institutional and international standards for research involving human subjects. This study utilized an anonymous online questionnaire with voluntary participation. Before accessing the survey, respondents reviewed an electronic information sheet detailing the study purpose, eligibility criteria, risks, benefits, and strict data protection measures. Electronic informed consent was obtained from each participant prior to survey entry. Respondents retained the right to withdraw at any stage of the process without consequence. No personally identifiable details were collected, and all research data were kept confidential, used solely for academic purposes, and deleted immediately after the data collection phase concluded.

## 3. Results

The survey collected sociodemographic information (gender, age, occupation, and marital status), as presented in Table 2. Age was reported in grouped categories (range = 18–28), with 46.77% aged 26–28 years, 36.29% aged 22–25 years, and 16.94% aged 18–21 years. The sample was predominantly composed of working individuals (36.29% new hires, 15.73% entrepreneurs, 10.48% part-timers/freelancers), followed by 31.05% undergraduate students and 6.45% falling into other/unclassified categories. Participants’ marital status comprised 93.55% single, 5.24% married, and 1.21% divorced.

This study also documented participants’ digital behavior and psychosocial background (daily screen time, frequency of exposure to HGC, digital platforms used, history of clinician-diagnosed psychiatric disorders, and religious practices), as presented in Table 3. Daily screen time was reported as >4 h (63.31%), 2–4 h (31.45%), and <2 h (5.24%). Frequency of HGC exposure in the past month was classified into: rarely (25.4%), sometimes (30.65%), a few times a week (26.21%), and almost every day (17.74%). The digital platforms used by participants varied across providers and formats. As respondents were allowed to select multiple platforms, the total frequency does not sum to 248. Instagram was the most frequently used platform (30.23%), followed by web search or AI-based queries (23.53%), TikTok (20.42%), YouTube (16.50%), YouTube Shorts (6.70%), and conventional hospital websites or healthcare applications (2.62%). Although most participants (75.40%) reported no history of clinician-diagnosed psychiatric disorders or past/current psychotic symptoms, 61 respondents disclosed a prior diagnosis. The most commonly reported conditions were depression (9.68%), generalized anxiety disorder (6.45%), somatic symptom presentations (4.84%), ADHD (2.02%), and PTSD (1.61%). Lastly, religious practice was reported as routine practice by 37.50% respondents, neutral by 31.05%, less frequent by 10.89%, and none/absent by 20.56%.

Construct validity and reliability for all latent variables are summarized in Table 4, which reports indicator loadings, internal consistency estimates, and convergent validity statistics for each construct. Cronbach’s alpha (CA = 0.769–0.910), rho_a, and rho_c all exceed recommended thresholds, while AVE values are above 0.50, indicating adequate internal consistency and convergent validity across all constructs. Most outer loadings meet or exceed the preferred 0.70 benchmark (for example, HMV, NRT, PEM, and NEU items all load strongly >0.80), supporting clear item–construct correspondence. A small number of indicators are marginal: DHL-1 at 0.698, and PWB-3 at 0.682, which are close to the 0.70 rule and acceptable given their contribution to overall scale reliability. In contrast, CYR-6 shows a weak loading (0.557) and reduces the indicator set for CYR. However, given the CYR scale’s high internal consistency (CA = 0.901, AVE = 0.683), CYR-6 was retained to preserve content validity, as it captures a distinct health-related cognitive worry component central to cyberchondria resilience and not represented by the remaining behavioral items. This also aligns with the methodological recommendations of Henseler et al. (2015), which find no indicators of discriminant validity issues for inter-construct correlations of 0.70 or less [90]. In sum, the psychometric evidence supports proceeding with the planned structural analyses.

To ensure that each construct represented a distinct concept, the next stage of validity testing examined discriminant validity. The evaluation was conducted using the heterotrait–monotrait (HTMT) ratio with bootstrapped 95% confidence intervals based on 10,000 resamples, as presented in Table 5. Two HTMT pairings exceeded the conservative 0.90 threshold, specifically NRT and HMV (HTMT = 0.928, 95% CI [0.870–0.979]), as well as PSR and PWB (HTMT = 0.927, 95% CI [0.882–0.965]). However, the 95% bootstrap intervals for both pairs exclude 1.00, indicating that these constructs remain statistically distinct. Nevertheless, the high HTMT values indicate considerable conceptual overlap and shared variance between these constructs. This proximity suggests that respondents perceive NRT/HMV and PSR/PWB as closely aligned concepts. As a result, interpretation of the structural paths should be viewed as part of a broader integrated framework. Although the constructs remain statistically distinct, their empirical similarity indicates that they function as interconnected components within the model rather than isolated ones. Other pairings showed elevated but subthreshold HTMT values, for example, ERS with PSR (HTMT = 0.870, 95% CI [0.788–0.942]) and NRT with ERS (HTMT = 0.829, 95% CI [0.740–0.902]); their confidence intervals likewise exclude 1.00. Overall, while the HTMT diagnostics confirm statistical discriminant validity across the measurement model, the elevated ratios for the NRT/HMV and PSR/PWB pathways highlight conceptually proximal nodes that function as complementary, reinforcing mechanisms rather than isolated, competing predictors within the structural model.

**Figure 3:**
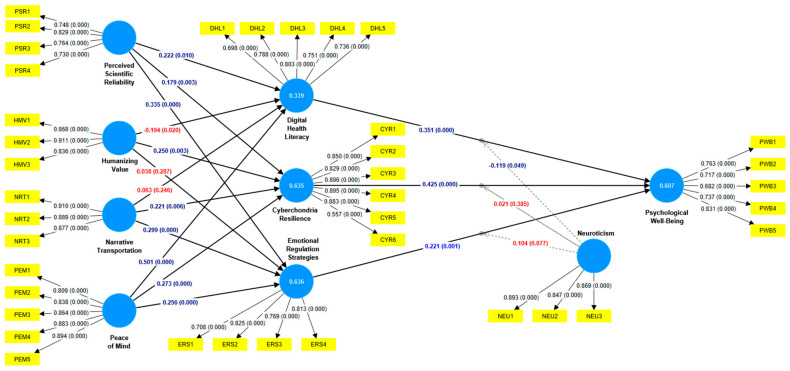
PLS–SEM structural model. Solid arrows represent structural paths and numbers denote path coefficients and R2 values.

Having established that the measurement model demonstrates sufficient reliability and validity, it is appropriate to proceed with the inferential testing of the hypothesized structural relationships. The inferential analysis with PLS-SEM was obtained from the results of percentile bootstrapping using 10,000 sub-samples with a one-tailed test and a significance level of 0.05. An overview of the interrelationships among the study constructs can be seen in Figure 3.

To evaluate the practical predictive utility of the proposed structural model, we executed the PLS predict procedure using a 10-fold cross-validation protocol [91]. The cross-validated predictive ability test (CVPAT) was used to evaluate whether PLS-SEM outperforms indicator-average (IA) and linear regression models (LM) in terms of prediction error, as summarized in Table 6. The CVPAT comparisons reveal that PLS-SEM yields lower out-of-sample prediction loss than the IA benchmark for all endogenous constructs (*p* < 0.001), confirming superior predictive validity. Compared with the LM, PLS-SEM delivered comparable performance overall, with modest but statistically significant advantages for cyberchondria resilience (*p* = 0.001) and emotional regulation strategies (*p* = 0.025), with no significant differences for digital health literacy or psychological well-being (*p* = 0.281 and *p* = 0.922). At the model level, PLS-SEM demonstrated superior predictive accuracy versus IA (*p* < 0.001) and equivalent accuracy to LM (*p* = 0.463), supporting its use for the study’s predictive objectives.

The bias-corrected standardized path coefficients for all hypothesized relationships can be seen in Table 7, including 95% bootstrap confidence intervals and *p*-values.

The construct of PSR demonstrated a consistent and strong positive association across mechanisms. It was significantly associated with DHL (β = 0.222, *p* = 0.010), CYR (β = 0.179, *p* = 0.003), and ERS (β = 0.335, *p* < 0.001). These findings indicate that trustworthy, evidence-based HGC, as perceived by young adults, associates with stronger cognitive and affective coping capacities. Humanizing value (HMV) showed a mixed pattern of associations, exhibiting a negative relationship with DHL (β = −0.194, *p* = 0.020) while maintaining a significant positive association with CYR (β = 0.250, *p* = 0.003). At the same time, its association with ERS was non-significant (β = 0.038, *p* = 0.287), suggesting that empathetic content relates to psychological safety but does not independently correspond with structured information-processing skills. Narrative transportation (NRT) was positively associated with both CYR (β = 0.221, *p* = 0.006) and ERS (β = 0.299, *p* < 0.001). Its relationship with DHL remained non-significant (β = 0.063, *p* = 0.246), indicating that immersive storytelling aligns with emotional adaptation without necessarily relating to stronger information-use competence. Peace of mind (PEM) emerged as the most prominent correlate within the model. It showed strong positive associations with DHL (β = 0.501, *p* < 0.001), CYR (β = 0.273, *p* < 0.001), and ERS (β = 0.256, *p* < 0.001). These results underscore the central role of psychological safety in relation to adaptive responses to health information. In the final structural paths, all three mediators showed significant positive associations with PWB. These included DHL (β = 0.351, *p* < 0.001), CYR (β = 0.425, *p* < 0.001), and ERS (β = 0.221, *p* = 0.001), with CYR demonstrating the strongest association with well-being. Neuroticism (NEU) moderated only the association between DHL and PWB, where higher levels of NEU corresponded with a weaker relationship between these variables (β = −0.119, *p* = 0.049). Moderation of the CYR and ERS pathways was not statistically significant, suggesting that these mechanisms remain relatively stable across different levels of baseline negative affectivity.

## 4. Discussion

### 4.1. General Discussion

This study evaluated an 18-hypothesis model investigating digital health literacy (DHL), cyberchondria resilience (CYR), and emotion regulation strategies (ERS) as mediators, and neuroticism (NEU) as a moderator, of psychological well-being (PWB) among Generation Z in Indonesia. The measurement model demonstrated consistent reliability and construct validity (Table 4), as well as predictive utility (Table 6), retaining marginal indicators like CYR-6 to preserve essential cognitive dimensions. Discriminant validity analyses (Table 5) confirmed that conceptually overlapping constructs operate as empirically distinct, reinforcing mechanisms rather than isolated predictors. Within the structural model, 13 hypotheses were supported (Table 7). Hypotheses H13 through H18 represent the primary direct pathways linking independent perceived HGC attributes to PWB. Testing these direct relationships is a methodological requirement to accurately assess the associative mediation exerted by DHL, CYR, and ERS. The remaining hypotheses serve a foundational role by establishing necessary links between content attributes and the mediators themselves, making it statistically possible to determine how viewer perceptions ultimately relate to overall well-being. Among these pathways, CYR emerged as the strongest mediator. Conversely, NEU acted as a significant moderating barrier that positions health information as a liability for vulnerable individuals. These findings indicate that highlighting perceived scientific reliability in hospital communications could be beneficial in supporting resilience and addressing vulnerabilities associated with health-anxious personality traits.

Perceived scientific reliability (PSR) produced positive effects across cognitive and affective pathways, improving DHL, strengthening CYR, and enhancing ERS. A plausible mechanism linking PSR to DHL operates through the incorporation of ‘credibility cues’, such as verifiable citations and nuanced medical terminology [92]. Repeated exposure to such content may train emerging adults to distinguish trustworthy from misleading health information, thereby strengthening evaluative and analytical skills [93]. Unlike sensationalist media, reliable content emphasizes the probabilistic nature of evidence-based medicine, framing individual findings as components of a broader knowledge base rather than absolute conclusions [94]. This framing may reduce catastrophic interpretation of health information and limit ruminative reassurance-seeking [95,96], supporting CYR. By limiting sensationalism and exaggerated claims [94,97], reliable content also fosters an environment where ERS can flourish. Rather than reacting impulsively to health threats, emerging adults can develop a sophisticated capacity to discern which information warrants a reactive response. This suggests a potential mediation effect where PSR is related to CYR through the development of DHL. Future studies should move away from linear models to investigate how these variables form a reciprocal network that protects Generation Z’s well-being.

Peace of mind (PEM) also demonstrated consistent associations with the psychological mediators, exerting its strongest effect on DHL. Evidence shows that high threat appraisal correlates significantly with reduced deep cognitive processing and fosters more avoidance or anxiety [98,99]. In this context, when young adults feel secure in the information they encounter, they may be less prone to maladaptive emotional escalation and compulsive checking behaviors. This emotionally regulated state supports more effective engagement with health information and reinforces resilience, underscoring peace of mind as a critical upstream determinant of both cognitive and affective outcomes.

Humanizing value (HMV) exhibited a divergent pattern, enhancing CYR while showing a negative association with DHL. This pattern suggests that compassion may successfully reduce distress and panic responses without cultivating information competence [100]. While reassurance attenuates anxiety in the short term, it may inadvertently foster passive reliance on institutional authority rather than autonomous information processing. Such findings are consistent with the understanding that short-term emotional relief does not reliably promote long-term self-regulatory or evaluative skills, particularly in health-anxious individuals [101,102]. Accordingly, humanizing content is protective for distress but insufficient on its own for promoting adaptive digital-health behaviors, underscoring the need to balance empathy with explicit literacy scaffolding to avoid reinforcing dependency or superficial understanding.

Narrative transportation (NRT) facilitated CYR and ERS, while it was insignificantly associated with DHL. This pattern may reflect the capacity of emotionally structured narratives to facilitate emotional exploration and meaning-making processes, thereby supporting adaptive coping, as demonstrated in young adults with cancer engaging with narrative content [103]. In contrast, immersive storytelling does not intrinsically engage evaluative or metacognitive operations, and without metacognitive prompts, storytelling boosts engagement without advancing critical appraisal or fact-checking proficiency [104].

All three psychological mediators were positively associated with psychological well-being, but cyberchondria resilience (CYR) emerged as the most influential pathway, underscoring the central role of anxiety regulation in shaping overall well-being. This stronger relationship likely stems from its direct influence on the affective and behavioral processes that modulate distress [105,106]. Unlike digital health literacy (DHL), which functions primarily as a cognitive information skill [85], CYR indexes tolerance of uncertainty and the cessation of compulsive reassurance seeking [105]. These components align fundamentally with clinical indicators of psychological resilience, including affective stability and preserved functioning [27]. Similarly, specific emotion regulation strategies represent only a limited subset of the broader protective repertoire found in resilience [107,108]. From a cognitive-behavioral viewpoint, resisting the maladaptive behaviors associated with cyberchondria yields more immediate gains in mood compared to analytic skill alone [108,109]. These results suggest that CYR is more closely linked to immediate adaptive outcomes, while other factors operate partially or distally. This finding therefore supports the value of clinical interventions that focus on reducing compulsive health checking to enhance adolescent mental health.

High levels of neuroticism appear to neutralize the positive association between DHL and PWB while leaving CYR and ERS pathways largely intact. This selective moderation likely occurs because neuroticism transforms digital health literacy into a source of worry by encouraging catastrophic appraisals of health information [69,110]. For these vulnerable individuals, the ability to search for information becomes a liability that fuels rumination rather than a skill that supports mental health [109]. In contrast, the CYR and ERS pathways remain comparatively unaffected, likely because they operate through mechanisms that directly target affective adjustment and behavioral coping, processes that are comparatively less dependent on cognitive appraisal biases and therefore remain comparatively unaffected across levels of neuroticism [111]. These results suggest that while informational interventions are context-dependent, affective strategies provide a stable foundation for well-being that is not easily compromised by negative personality traits [112].

To our knowledge, this study is the first to systematically examine the relationship between perceived hospital-generated content characteristics and psychological well-being in Generation Z through interconnected cognitive and emotional pathways, with neuroticism moderating these effects. These results establish a conceptual foundation for the development of multifaceted mental health strategies suited to the digital age. Future research should employ longitudinal and experimental designs to validate the causal impact of integrating credibility cues with affective reassurance. The inconsistent findings regarding narrative transportation and humanizing values indicate that these elements may require more nuanced application within developmental psychology. Expanding this research through cross-cultural studies will clarify the role of institutional trust in shaping digital resilience. Additionally, further psychometric refinement of the cyberchondria resilience scale is essential to establish its utility as a primary outcome measure in clinical studies.

### 4.2. Practical Implications for Healthcare Providers

Mental disorders impose substantial personal, social, and economic burdens [113]. Addressing these challenges requires a shift from clinical symptom management toward optimizing the digital ecosystems in which patients actually live and process health threats. Among social determinants amenable to intervention, digital health directly contributes to environmental safety by shaping the information ecology [114]. This analysis provides a framework to leverage institutional health communication as a legitimate preventive tool. By targeting the intersection of affective processing and digital information, clinicians can effectively de-escalate psychological vulnerability within the young adult community and promote autonomous health management through evidence-based digital design.

Given that anxiety disorders affect approximately 4.9% of adolescents and young adults (particularly Generation Z) globally [115], early prevention of digitally mediated health anxiety or somatization is a public health imperative. This urgency is underscored by established evidence identifying neuroticism as a primary personality trait that increases susceptibility to internalizing disorders such as depression and anxiety [67]. Even among Generation Z individuals currently categorized as mentally well, the absence of a formal psychopathology does not equate to a lack of risk. Mental health professionals are therefore encouraged to adopt an upstream approach, moving beyond reactive crisis management to prevent sub-clinical distress from crossing diagnostic thresholds, as mental health promotion and prevention remains a fundamental clinical objective [57,113].

The findings of this study offer a roadmap to reorient institutional digital communication for early prevention and community intervention. Cyberchondria resilience emerged as a pivotal protective factor, implying that reducing anxiety-driven health behaviors may yield broader benefits for psychological well-being than literacy alone. These findings align with meta-analytic evidence among young adults emphasizing that risk-stratified digital interventions can benefit well-being and mitigate ill-being symptoms [116]. Young adults with high trait neuroticism may experience high volumes of medical information as overwhelming, potentially exacerbating catastrophic thinking [72]. In this context, focusing on peace of mind could help maintain the role of literacy as a protective factor. This aligns with evidence that suggests emotion regulation is a prerequisite for effective cognitive engagement during adolescence and emerging adulthood [117]. By integrating scientific reliability with specific cognitive behavioral therapy (CBT) and dialectical behavioral therapy (DBT) principles, Indonesian healthcare institutions could potentially support the development of cognitive buffers against ruminative anxiety. For example, content may incorporate exposure and response prevention (ERP) prompts that encourage users to delay compulsive searching [118,119]. This behavioral strategy is hypothesized to operate in parallel with DBT-informed mindfulness speed bumps designed to help manage emotional arousal before the presentation of complex medical data [120].

By incorporating a CBT-informed approach with clear signals of evidence and probabilistic framing, hospital-generated content may encourage emerging adults to check information thoughtfully and treat uncertainty as something that can be managed. This strategy aligns with meta-analytic evidence demonstrating that CBT produces reliable benefits in quality of life and mental health across diverse populations [121]. However, facilitating this level of sophisticated content appraisal is best supported by the involvement of psychiatrists, clinical psychologists, counselors, and digital mental health teams. These professionals are uniquely equipped to collaborate on translating cognitive-behavioral principles into digital practice that aligns with local socio-cultural norms. A biopsychosocial perspective further assists Indonesian practitioners in identifying specific temperamental risks that appear to be aggravated by digital health information [122]. By participating in content design, clinicians can contribute to digital environmental engineering to address systemic contributors to young adult anxiety. Therefore, it may be beneficial for Indonesian healthcare institutions to consult their respective mental health professionals to integrate clinical insights directly into institutional communication workflows. This collaborative screening process can ensure that the nation’s health content is clinically optimized to foster the “Wise Mind” state [123] relevant to autonomous health management [124].

Furthermore, the “comfort-without-competence” mechanism identified in this study suggests a potential risk in over-reliance on purely humanizing or compassionate messaging [100]. Warm, humanizing language may effectively reduce short-term distress, but without explicit skills training, it runs the risk of cultivating passive dependency on institutional authority. Empathy alone appears to be insufficient for building long-term resilience. To promote autonomy, a balanced communicative approach that pairs affective containment with explicit critical appraisal cues could be highly beneficial [125]. This combined framework is consistent with digital messaging that is designed to encourage independent coping strategies rather than promoting passive reliance.

This professional approach is intended to assist emerging adults in transitioning from passive reassurance-seeking to autonomous information processing, potentially strengthening the cognitive and affective capacities required for lifelong mental well-being. Recognizing digital platforms as potential sites for psychological prevention could assist healthcare systems in reducing community mental health burdens while equipping emerging adults with tools for navigating health uncertainty. To optimize this effort, it may be advantageous for Indonesian hospitals to collaborate with a broad network of stakeholders, including government agencies and educational institutions, to help cultivate an ecosystem that prioritizes effective mental health advocacy. This collaborative framework aims to support the development of accessible content that is linked with long-term psychological stability and empowers youth to manage health-related stress safely.

### 4.3. Study Limitations

This study identifies several limitations that should be considered when interpreting the results. The cross-sectional study design prevents claims of causality, restricting the interpretation of these relationships to associative pathways. Despite implementing protective measures against CMB, data collection relied on self-reports and open-ended questions, leaving the data susceptible to common method variance. Furthermore, examining the relationships between these variables in a homogeneous population provides meaningful insights into the chosen specific age (18–29) and culture group (Indonesian) but limits the accuracy of their interpretation across populations. Therefore, expanding the population criteria in future studies is recommended to provide a basis for comparison across different age groups and cultures.

## 5. Conclusions

The present study concludes that the mental health of Generation Z in Indonesia is associated with various health content-related variables. Specifically, a positive perception of hospital-generated content appears to support a trajectory toward well-being, partially by facilitating a cyberchondria resilience pathway. This dynamic positions hospital-generated content as a potential social-cognitive determinant of mental health. By establishing cyberchondria resilience as the primary mediator, these results suggest the potential value of a clinical shift toward addressing maladaptive health-seeking patterns rather than focusing solely on digital health literacy. While perceived scientific reliability and peace of mind provide essential protective buffers, the “comfort-without-competence” trap warns that affective containment unpaired with explicit cognitive scaffolding might induce dependency. Furthermore, the paradoxical associations of digital health literacy underscore the importance of stratified approaches that prioritize emotion regulation over information density to help prevent health information from triggering ruminative anxiety.

Translating these findings into practice, Indonesian healthcare systems could explore a “psychological vaccine” model by integrating CBT and DBT principles within their digital platforms. Employing credibility cues along with mindfulness cues (e.g., speed bumps, cues to slow down information processing) could help protect vulnerable youth from the risks of harmful rumination cycles and promote a “Wise Mind” state linked with health autonomy. Accomplishing this highly collaborative effort would likely benefit from active partnerships among healthcare providers, government agencies, and educational institutions, rooted in a shared vision and motivation, to support an integrated ecosystem for mental health advocacy. This framework conceptualizes digital health communication as a potentially powerful strategy for community-based prevention, offering pathways to support emerging adults in navigating the uncertainties of today’s health information landscape with sustainable cognitive and affective resilience.

## Figures and Tables

**Table 1:** Conceptualized definition of constructs.

Variables	Conceptualized Definition	References
Perceived Scientific Reliability	The extent to which individuals interpret HGC as factually accurate, evidence-based, verifiable, and delivered by a trustworthy source that communicates transparently and corrects errors responsibly.	[39]
Humanizing Value	The extent to which HGC fosters sensorial, emotional, intellectual, and behavioral experiences that feel personal, empathetic, and relational rather than institutional.	[45]
Narrative Transportation	The extent to which individuals become cognitively, emotionally, and imaginatively immersed in HGC, experiencing focused mental engagement, enduring affective involvement, and vivid visualization of the scenarios depicted.	[49]
Peace of Mind	The sense of security and calm that arises when individuals perceive HGC as trustworthy, easy to understand, and clearly intended to protect their health interests.	[54,55]
Cyberchondria Resilience	The ability to manage and recover from health-related anxiety induced by online health information, appropriate control over information-seeking behavior, emotional stability during health-related uncertainty, and discern credible medical content without seeking forced reassurance.	[5]
Digital Health Literacy	An individual’s ability to effectively locate, evaluate, and apply health information from hospital-generated digital platforms, enabling informed health-related decisions through critical assessment and practical use of online medical content.	[85]
Emotional Regulation Strategies	The deliberate use of cognitive reappraisal and expressive suppression to manage, modify, or control the intensity and expression of emotional experiences.	[86]
Neuroticism	A personality trait reflecting an individual’s predisposition toward emotional instability, negative affectivity, and heightened reactivity to stress.	[87]
Psychological Well-Being	The subjective experience of frequent positive emotions, low negative affect, and favorable self-evaluations of one’s mental state, emphasizing the quality of emotional life as a dynamic condition that varies across individuals and can be shaped by situational contexts and adaptive coping processes.	[27,28,58]

**Table 2:** Respondents’ sociodemographic profile.

Categories	Description	Amount (*n*)	Percentage (%)
Gender	Male	85	34.27
	Female	151	60.89
	Prefer not to answer	12	4.84
Age	26–28	116	46.77
	22–25	90	36.29
	18–21	42	16.94
Occupation	Undergraduate students	77	31.05
	New hires	90	36.29
	Entrepreneurs	39	15.73
	Part-timers/freelancers	26	10.48
	Others	16	6.45
Marital Status	Single	232	93.55
	Married	13	5.24
	Divorced	3	1.21

**Table 3:** Respondents’ behavioral and psychosocial characteristics.

Categories	Description	Amount (*n*)	Percentage (%)
Daily screen time	<2 h	13	5.24
	2–4 h	78	31.45
	>4 h	157	63.31
HGC exposure	Rarely	63	25.40
	Sometimes	76	30.65
	A few times a week	65	26.21
	Almost every day	44	17.74
Digital platforms	Instagram	185	30.23
	Tiktok	125	20.42
	Youtube Shorts	41	6.70
	Youtube	101	16.50
	Web search/AI query	144	23.53
	Hospital websites/Healthcare applications	16	2.62
	Total	612	100
History of known psychiatric diagnosis	Depression	24	9.68
	Generalized Anxiety Disorder (GAD)	16	6.45
	Somatic symptoms	12	4.84
	Post-Traumatic Stress Disorder (PTSD)	4	1.61
	Attention-Deficit/Hyperactivity Disorder (ADHD)	5	2.02
	None	187	75.40
Religious practice	Routinely	93	37.50
	Neutral	77	31.05
	Less frequent	27	10.89
	None	51	20.56

**Table 4:** Construct reliability and validity.

Variable	Code	Indicator	OL
Perceived Scientific Reliability	PSR-1	The information presented by the HGC is reliable and reflects accurate interpretations of medical knowledge.	0.748
	PSR-2	The information from HGC is supported by verifiable scientific sources and transparent references.	0.829
	PSR-3	HGC uses the latest and most credible scientific evidence.	0.764
	PSR-4	HGC is transparent and not intended to mislead or overstate medical claims.	0.730
	Mean = 4.107, CA = 0.769, rho_a = 0.769, rho_c = 0.852, AVE = 0.591		
Humanizing Value	HMV-1	HGC makes me feel that the hospital genuinely cares about people like me.	0.868
	HMV-2	HGC evokes a sense of compassion and empathy.	0.911
	HMV-3	HGC encourages me to take an active part in maintaining my own health.	0.836
	Mean = 4.162, CA = 0.842, rho_a = 0.845, rho_c = 0.905, AVE = 0.761		
Narrative Transportation	NRT-1	While viewing HGC, I could clearly imagine the real-life scenarios it depicted.	0.910
	NRT-2	After finishing HGC, it stayed on my mind.	0.889
	NRT-3	I was immersed in HGC while reading or viewing it.	0.877
	Mean = 4.074, CA = 0.871, rho_a = 0.874, rho_c = 0.921, AVE = 0.795		
Peace of Mind	PEM-1	I feel confident in the hospital’s medical expertise and the accuracy of the information they share.	0.809
	PEM-2	The hospital makes it easy for me to access and understand their health information, which helps me feel secure.	0.838
	PEM-3	HGC reassures me that they will support my well-being in the long term.	0.864
	PEM-4	Because I already trust the hospital, I feel genuinely cared for through the content they provide.	0.883
	PEM-5	Engaging with HGC makes me feel enlightened.	0.894
	Mean = 4.107, CA = 0.910, rho_a = 0.910, rho_c = 0.933, AVE = 0.736		
Cyberchondria Resilience	CYR-1	I am able to manage feelings of anxiety or distress after consuming health information from HGC.	0.850
	CYR-2	I can minimize panic when I come across HGC that links common symptoms to severe illnesses.	0.829
	CYR-3	I can resist the urge to revisit the same HGC or search the same symptoms multiple times online.	0.896
	CYR-4	I can ensure that looking up hospital-generated health information does not interfere with my focus at work, study, or other online activities.	0.895
	CYR-5	I can remain calm and resist the urge to consult medical professionals for reassurance after consuming HGC.	0.883
	CYR-6	I can manage worries that something might be wrong with my body after consuming HGC.	0.557
	Mean = 4.162, CA = 0.901, rho_a = 0.909, rho_c = 0.927, AVE = 0.683		
Digital Health Literacy	DHL-1	I know how to find useful health information from hospital-generated digital platforms (e.g., websites, social media, apps).	0.698
	DHL-2	I know how to use digital HGC to answer my health-related questions.	0.788
	DHL-3	I know how to apply health information from hospital content to support my daily activities or health decisions.	0.803
	DHL-4	I can evaluate whether hospital-generated health information I find online is trustworthy.	0.751
	DHL-5	I can differentiate between correct and incorrect hospital-generated health information found on digital platforms.	0.736
	Mean = 3.727, CA = 0.812, rho_a = 0.811, rho_c = 0.870, AVE = 0.572		
Emotional Regulation Strategies	ERS-1	I keep my emotions to myself.	0.708
	ERS-2	I control my emotions by not expressing them.	0.825
	ERS-3	When I want to feel more positive emotion, I change the way I’m thinking about the situation.	0.769
	ERS-4	I control my emotions by changing the way I think about the situation I’m in.	0.813
	Mean = 3.979, CA = 0.785, rho_a = 0.798, rho_c = 0.861, AVE = 0.608		
Neuroticism	NEU-1	I tend to feel depressed or blue.	0.893
	NEU-2	I worry a lot.	0.847
	NEU-3	I am easily discouraged.	0.869
	Mean = 4.125, CA = 0.843, rho_a = 0.891, rho_c = 0.903, AVE = 0.756		
Psychological Well-Being	PWB-1	Over the past 2 weeks, I have felt cheerful and in good spirits.	0.763
	PWB-2	Over the past 2 weeks, I have felt calm and relaxed.	0.717
	PWB-3	Over the past 2 weeks, I have felt active and vigorous.	0.682
	PWB-4	Over the past 2 weeks, I woke up feeling fresh and rested.	0.737
	PWB-5	Over the past 2 weeks, my daily life has been filled with things that interest me.	0.831
	Mean = 3.942, CA = 0.803, rho_a = 0.815, rho_c = 0.863, AVE = 0.559		

OL = Outer loading, CA = Cronbach’s alpha, AVE = Average variance extracted.

**Table 5:** Discriminant validity with HTMT ratio.

**Variable**	**CYR**	**DHL**	**ERS**	**HMV**	**NRT**	**NEU**	**PEM**	**PSR**	**PWB**
CYR									
DHL	0.396 (0.258–0.525)								
ERS	0.803 (0.720–0.870)	0.537 (0.405–0.648)							
HMV	0.816 (0.737–0.881)	0.375 (0.213–0.527)	0.783 (0.689–0.860)						
NRT	0.801 (0.723–0.864)	0.446 (0.279–0.588)	0.829 (0.740–0.902)	0.928 (0.870–0.979)					
NEU	0.515 (0.405–0.616)	0.129 (0.062–0.185)	0.514 (0.382–0.626)	0.466 (0.326–0.590)	0.458 (0.319–0.574)				
PEM	0.770 (0.687–0.837)	0.636 (0.505–0.742)	0.801 (0.714–0.872)	0.745 (0.646–0.828)	0.772 (0.665–0.850)	0.388 (0.242–0.524)			
PSR	0.746 (0.646–0.827)	0.540 (0.420–0.641)	0.870 (0.788–0.942)	0.732 (0.629–0.815)	0.690 (0.581–0.779)	0.541 (0.417–0.653)	0.714 (0.619–0.798)		
PWB	0.779 (0.691–0.849)	0.670 (0.519–0.788)	0.778 (0.679–0.858)	0.642 (0.526–0.739)	0.739 (0.628–0.828)	0.299 (0.169–0.436)	0.927 (0.882–0.965)	0.653 (0.555–0.735)	

Notes: values in parentheses represent the 5–95% confidence interval. CYR = cyberchondria resilience, DHL = digital health literacy, ERS = emotional regulation strategies, HMV = humanizing value, NRT = narrative transportation, NEU = neuroticism, PEM = peace of mind, PSR = perceived scientific reliability, PWB = psychological well-being.

**Table 6:** Cross-validated predictive ability test (CVPAT).

Variable/Model	PLS-SEM vs. Indicator Average (IA)				PLS-SEM vs. Linear Model (LM)			
	PLS Loss	IA Loss	Average Loss Difference	*p*-Value	PLS Loss	LM Loss	Average Loss Difference	*p*-Value
Digital Health Literacy	0.544	0.649	−0.105	0.001	0.544	0.522	0.023	0.281
Cyberchondria Resilience	0.195	0.320	−0.125	0.000	0.195	0.218	−0.022	0.001
Emotional Regulation Strategies	0.231	0.358	−0.127	0.000	0.231	0.249	−0.018	0.025
Psychological Well-Being	0.323	0.458	−0.134	0.000	0.323	0.325	−0.001	0.922
Overall/Model	0.322	0.444	−0.123	0.000	0.322	0.327	−0.005	0.463

**Table 7:** Standardized path coefficient for path analysis (bias-corrected).

Path	Std. Pathcoefficient (β)	*p*-Value	Confidence Interval (CI)		Interpretation
			5%	95%	
PSR → DHL (H1)	0.222	0.010	0.059	0.371	Significant positive effect
PSR → CYR (H2)	0.179	0.003	0.074	0.286	Significant positive effect
PSR → ERS (H3)	0.335	0.000	0.240	0.429	Significant positive effect
HMV → DHL (H4)	−0.194	0.020	−0.348	−0.040	Significant, but negative effect
HMV → CYR (H5)	0.250	0.003	0.106	0.405	Significant positive effect
HMV → ERS (H6)	0.038	0.287	−0.077	0.145	Not significant, positive effect
NRT → DHL (H7)	0.063	0.246	−0.092	0.210	Not significant, positive effect
NRT → CYR (H8)	0.221	0.006	0.074	0.359	Significant positive effect
NRT → ERS (H9)	0.299	0.000	0.176	0.431	Significant positive effect
PEM → DHL (H10)	0.501	0.000	0.356	0.640	Significant positive effect
PEM → CYR (H11)	0.273	0.000	0.157	0.379	Significant positive effect
PEM → ERS (H12)	0.256	0.000	0.143	0.361	Significant positive effect
DHL → PWB (H13)	0.351	0.000	0.235	0.465	Significant positive effect
CYR → PWB (H14)	0.425	0.000	0.309	0.553	Significant positive effect
ERS → PWB (H15)	0.221	0.001	0.104	0.344	Significant positive effect
NEU × DHL → PWB (H16)	−0.119	0.049	−0.233	0.001	Significant negative effect
NEU × CYR → PWB (H17)	0.021	0.385	−0.101	0.130	Not significant, positive effect
NEU × ERS → PWB (H18)	0.104	0.077	−0.004	0.232	Not significant, positive effect

PSR = perceived scientific reliability, DHL = digital health literacy, CYR = cyberchondria resilience, HMV = humanizing value, ERS = emotional regulation strategies, NRT = narrative transportation, PEM = peace of mind, NEU = neuroticism, PWB = psychological well-being.

## Data Availability

The data from this study are available on request due to privacy and ethical reasons.

## Abbreviations

The following abbreviations are used in this manuscript:

ADHD	Attention-Deficit/Hyperactivity Disorder
AVE	Average Variance Extracted
CA	Cronbach’s Alpha
CBT	Cognitive Behavioral Therapy
CI	Confidence Interval
CMB	Common Method Bias
CVPAT	Cross-Validated Predictive Ability Test
CYR	Cyberchondria Resilience
DBT	Dialectical Behavioral Therapy
DHL	Digital Health Literacy
ERS	Emotional Regulation Strategies
GAD	Generalized Anxiety Disorder
HGC	Hospital-Generated Content
HMV	Humanizing Value
HTMT	Heterotrait–Monotrait Ratio
IA	Indicator Average
LM	Linear Model
NEU	Neuroticism
NRT	Narrative Transportation
OL	Outer Loading
PEM	Peace of Mind
PLS-SEM	Partial Least Squares Structural Equation Modeling
PSR	Perceived Scientific Reliability
PTSD	Post-Traumatic Stress Disorder
PWB	Psychological Well-Being
rho_a	Dijkstra–Henseler’s rho
rho_c	Composite Reliability
SD	Standard Deviation
WHO	World Health Organization
